# Proanthocyanidins from Grape Seeds Modulate the NF-*κ*B Signal Transduction Pathways in Rats with TNBS-Induced Ulcerative Colitis

**DOI:** 10.3390/molecules16086721

**Published:** 2011-08-08

**Authors:** Xiaoli Li, Xiaolai Yang, Yongqing Cai, Hong Qin, Li Wang, Yanhong Wang, Yanhui Huang, Xiaoxia Wang, Shuai Yan, Liping Wang, Xin Zhao, Wan Li, Sijia Li, Jiajia Chen, Yongjie Wu

**Affiliations:** 1 Key Laboratory of Preclinical Study for New Drugs of Gansu Province, Department of College of Medicine, School of Basic Medical Sciences, Lanzhou University, Lanzhou 730000, China; 2 People’s Hospital of Gansu Province, Lanzhou 730000, China; 3 Department of Pharmacology, College of Medicine, The Third Military Medical University, Chongqing 400038, China; 4 Department of Pharmacy, Southwestern Hospital, The Third Military Medical University, Chongqing 400038, China

**Keywords:** proanthocyanidins from grape seeds, ulcerative colitis, nuclear factor-kappa B

## Abstract

Abstract To elucidate the molecular mechanisms involved in the therapeutic effects of proanthocyanidins from grape seeds (GSPE), we explore whether GSPE regulates the inflammatory response of TNBS-induced colitis in rats at the levels of NF-*κ*B signal transduction pathway. Rats were intragastrically administered of different doses of GSPE (100, 200 and 400 mg·kg^−1^) per day for seven days after ulcerative colitis (UC) was induced by intracolonic injection of 2,4,6-trinitrobenzenesulfonic acid (TNBS) dissolved in 50% ethanol. Sulfasalazine (SASP) at 400 mg/kg was used as a positive control drug. The expression of nuclear factor-kappa B (NF-*κ*B), phospho-I kappaB-alpha (pI*κ*Bα), inhibitor kappa B kinase (I*κ*K) in the colon tissues were all measured by enzyme-linked immunosorbent assay (ELISA) methods. Treatment with GSPE reduced the expression of NF-*κ*B, pI*κ*Bα and I*κ*K in the colon. The results of this study show that GSPE exerts beneficial effects in inflammatory bowel disease by inhibition of NF-*κ*B signal transduction pathways.

## 1. Introduction

Inflammatory bowel diseases (IBDs), such as ulcerative colitis (UC) and Crohn’s disease (CD) [1,2], are characterized by tissue edema and associated with chronic relapsing inflammation of the intestinal tract of unknown etiology [3]. Several agents used in the management of IBD, such as corticosteroids, sulfasalazine, and 5-aminosalicylic acid, have documented regulation of NF-*κ*B function [4]. Intramuscular administration of proanthocyanidins from grape seeds (GSPE) has a strong anti-inflammatory activity [5,6]. Pharmacokinetic studies have confirmed that degradation occurs mainly in the colon. This metabolic characteristic suggests that GSPE affects the colonic mucosa directly, so that it has a natural colon-targeting feature that may be of therapeutic interest in UC. Our previous study has demonstrated that GSPE exerts a beneficial anti-inflammatory effect in the acute phase of TNBS-induced colitis in rats by downregulation some of the mediators involved in the intestinal inflammatory response, inhibiting inflammatory cell infiltration and antioxidation damage, promoting damaged tissue repair to improve colonic oxidative stress, decreasing production of proinflammatory cytokines IL-1β, and increasing production of antiinflammatory cytokines IL-4 [7]. Further study has shown that GSPE was effective on recurrent colitis [8]. These provide favorable evidence that GSPE may be useful for the treatment of UC in a clinical setting. However, the pivotal elements of its inhibitory action on the inflammation remain unclear. Important candidates are transcription factors that bind to gene promoter regions and are involved in the regulation of inflammation gene transcription. These potent activities of GSPE may be attributed to regulate some of certain signal transduction pathways critical to inflammatory responses, such as NF-*κ*B.

NF-*κ*B controls many important biological decisions, from formation of dorsal-ventral polarity in insects to activation of inflammatory and innate immune responses. The key event in NF-*κ*B activation is I*κ*B phosphorylation, the next logical step is to search for a stimulus-responsive I*κ*B kinase (I*κ*K) that could catalyze this event. The activated I*κ*K complexes then phosphorylate I*κ*B subunits in NF-*κ*B: I*κ*B complexes, triggering their ubiquitin-dependent degradation, and the activation of NF-*κ*B [9]. The objective of this study is to examine the role of NF-*κ*B as a causative mediator in modulation of experimentally induced colitis in the rat.

## 2. Results and Discussion

### 2.1. Colonic Microscopic Damage

**Figure 1:**
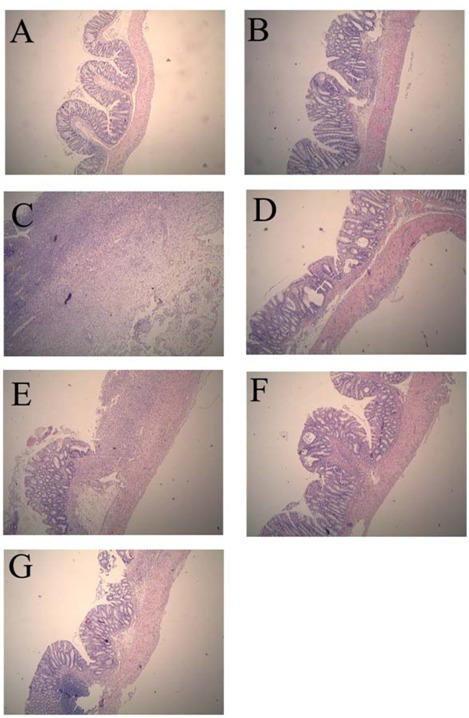
Effects of *proanthocyanidins from grape seeds* (GSPE) on microscopic of 2,4,6-trinitrobenzenesulfonic acid (TNBS)-induced rat colitis (original magnification, ×40). n = 12–14 per group. (**A**). normal control group; (**B**). 50% Ethanol control group showing oedema of the mucosa; (**C**). TNBS control group showing complete ulceration of the mucosa, oedema and intense diffuse transmural inflammatory infiltrate; (**D**). SASP group (500 mg/kg); (**E**). GSPE-L (100 mg/kg); (**F**). GSPE-M (200 mg/kg) and (**G**). GSPE-L (400 mg/kg) showing focal denudation with the presence of granulation tissue near well-preserved mucosa which, in turn, shows a loss of goblet cells.

Compared with the normal control group, histological assessment of the colonic specimens from the TNBS control group revealed mucosal damage, which was accompanied by goblet cell depletion, severe transmural inflammation and disruption of the normal architecture of the colon. The histological sections of GSPE treatment groups showed a progressive restoration, partially re-epithelialized, an improvement of the glandular structure and the level of congestion and edema reduction (Figure 1).

### 2.2. MPO Activity and IL-1β Levels in Colon

**Figure 2:**
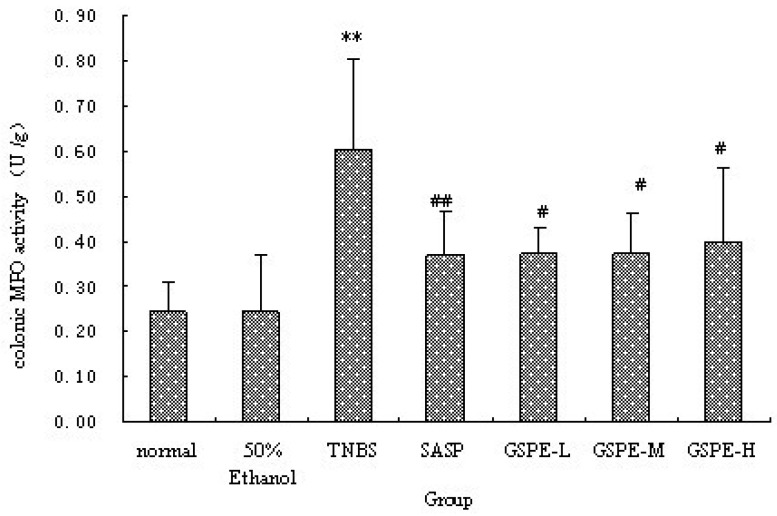
Colonic MPO activity after treatment with three doses of GSPE in rats with TNBS-induced colitis. Data were means ± SD, n = 12–14 per group. ** Significant at *P* < 0.01 *vs*. normal control group. ^#^ *P* < 0.05 and ^##^ *P* < 0.01 *vs*. TNBS control group (One-way ANOVA test). MPO: Myeloperoxidase; GSPE: Proanthocyanidins from grape seeds at GSPE-L 100 mg/kg, GSPE-M 200 mg/kg, or GSPE-H 400 mg/kg; TNBS:2,4,6-trinitrobenzenesulfonic acid; SASP: Sulfasalazine.

**Figure 3:**
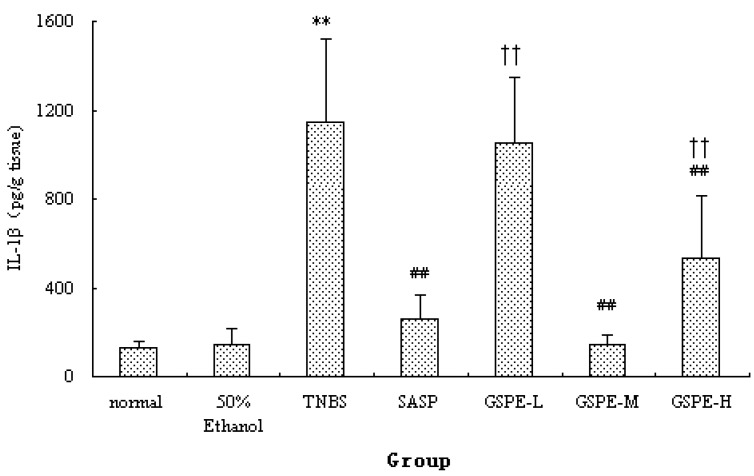
Colonic IL-1β levels after treatment with three doses of GSPE in rats with TNBS-induced colitis. Data were means ± SD, n = 12–14 per group. ** Significant at *P* < 0.01 *vs.* normal control group. ^##^ *P* < 0.01 *vs.* TNBS control group. ^††^ *P* < 0.01 *vs.* GSPE-M group (One-way ANOVA test). IL-1β: Interleukin-1β; GSPE: Proanthocyanidins from grape seeds at GSPE-L 100 mg/kg, GSPE-M 200 mg/kg, or GSPE-H 400 mg/kg; TNBS: 2,4,6-trinitrobenzenesulfonic acid; SASP, Sulfasalazine.

The colonic inflammation induced by TNBS was characterized by increased levels of colonic MPO and IL-1β. When GSPE or SASP was administered to colitic rats, a significant reduction in colonic MPO activity and IL-1β levels were observed in comparison with TNBS control group (Figure 2 and Figure 3). Howere, there was a significant difference between the different GSPE on IL-1β levels, IL-1β of the medium dose of GSPE was the lowest.

### 2.3. The Expression of Colonic IκK and PIκBα

**Figure 4:**
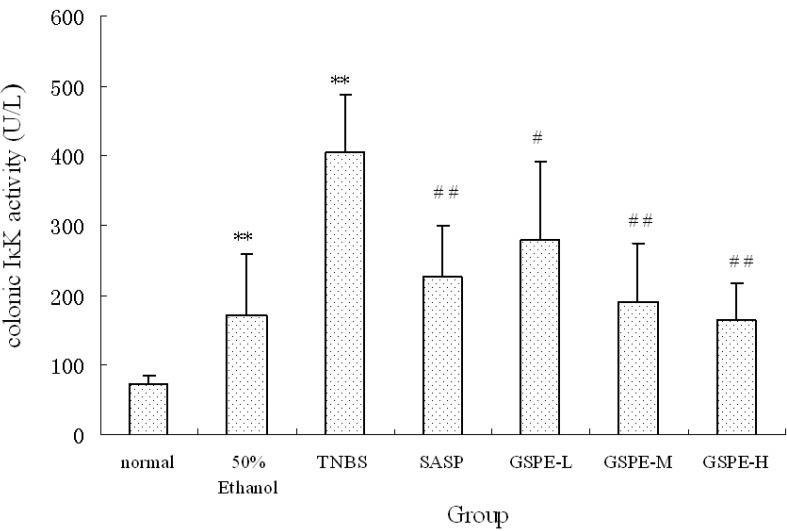
Colonic I*κ*K expression after treatment with three doses of GSPE in rats with TNBS-induced colitis. Data were means ± SD, n = 12–14 per group. ** Significant at *P* < 0.01 *vs.* normal control group. ^#^ *P* < 0.05 and ^##^ *P* < 0.01 *vs.* TNBS control group (One-way ANOVA test). I*κ*K: Inhibitor kappa B kinase; GSPE: Proanthocyanidins from grape seeds at GSPE-L 100 mg/kg, GSPE-M 200 mg/kg, or GSPE-H 400 mg/kg; TNBS: 2,4,6-trinitrobenzenesulfonic acid; SASP: Sulfasalazine.

**Figure 5:**
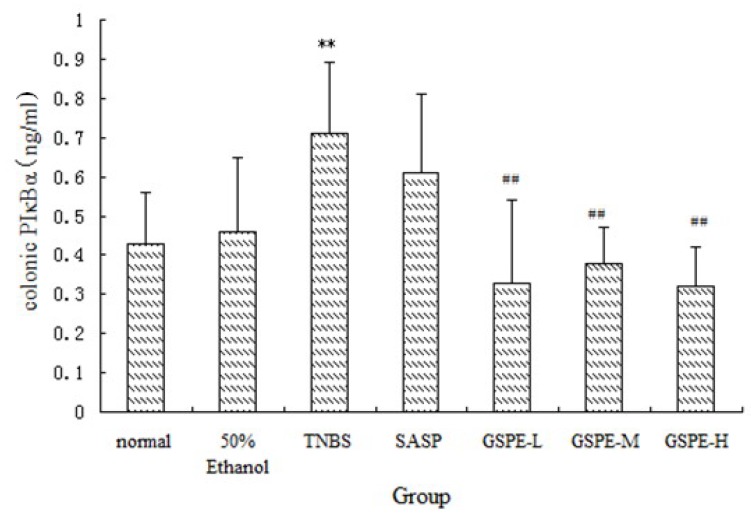
Colonic pI*κ*Bα expression after treatment with three doses of GSPE in rats with TNBS-induced colitis. Data were means ± SD, n = 12–14 per group. ** Significant at *P* < 0.01 *vs.* normal control group. ^#^ *P* < 0.05 and ^##^ *P* < 0.01 *vs.* TNBS control group (One-way ANOVA test). pI*κ*Bα: Phospho-I kappaB-alpha; GSPE: Proanthocyanidins from grape seeds at GSPE-L 100 mg/kg, GSPE-M 200 mg/kg, or GSPE-H 400 mg/kg; TNBS: 2,4,6-trinitrobenzenesulfonic acid; SASP: Sulfasalazine.

A significant increase of I*κ*K and PI*κ*Bα expression in cytoplasm of the TNBS control group compared with the normal control group. Compared with those of the TNBS control group, the expression of I*κ*K and PI*κ*Bα in the 3 GSPE treatment groups and SASP group were decreased (Figure 4 and Figure 5), but the decreased expression of PI*κ*Bα in the SASP group did not reach significance in comparison with the TNBS control group (Figure 5).

### 2.4. The Expression of Colonic NF-κB

**Figure 6:**
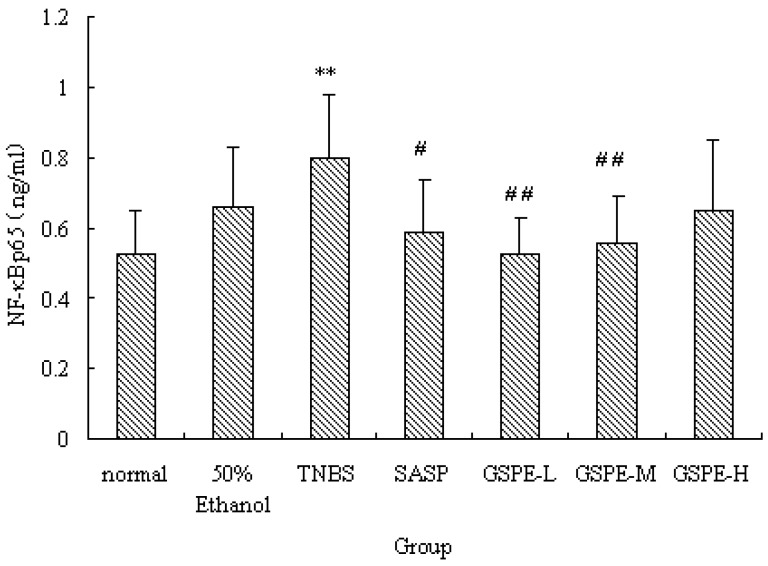
NF-*κ*B p65 expression of nuclear extracts after treatment with three doses of GSPE in rats with TNBS-induced colitis. Data were means ± SD, n = 12–14 per group. ** Significant at *P* < 0.01 *vs.* normal control group. ^#^ *P* < 0.05 and ^##^ *P* < 0.01 *vs.* TNBS control group (One-way ANOVA test). NF-*κ*B: Nuclear factor-kappa B; GSPE: Proanthocyanidins from grape seeds at GSPE-L 100 mg/kg, GSPE-M 200 mg/kg, or GSPE-H 400 mg/kg; TNBS: 2,4,6-trinitrobenzenesulfonic acid; SASP: Sulfasalazine.

NF-*κ*B is a heterodimer complex of p50 and p65 subunits and plays a central role in various inflammatory responses. On exposure to various inflammatory stimuli, NF-*κ*B is activated and translocates from the cytoplasma to the nucleus, where it then regulates the expression of various proinflammation cytokines such as IL-1, MPO. In our study, we measured NF-*κ*B p65 of nuclear extracts. Rats in the TNBS control group exhibited higher levels of NF-*κ*B p65 than those in the normal control group. However, the NF-*κ*B p65 levels were reduced by GSPE treated in a dose-dependent manner, compared with the TNBS control group, the NF-*κ*B p65 levels in the GSPE-L, GSPE-M and SASP groups were obviously down-regulated (Figure 6).

### 2.5. Discussion

It was previously demonstrated that GSPE protects against TNBS-induced colonic damage in rats. The protective effect of GSPE is probably associated with reducing granulocyte infiltration and decreasing the production of proinflammatory cytokine IL-1β in the colon of rats, in addition to its antioxidant effects [7].

NF-*κ*B is a crucial transcription factor which mediates transcriptional activation of many inflammatory genes. In the normal physiological state, NF-*κ*B exists in the cytoplasma as a heterodimer complex of p65/p50 subunits combined with an inhibitory protein, I*κ*B. Inflammatory stimulation provokes rapid degradation of I*κ*B, and subsequently the free NF-*κ*B molecule translocates into the nucleus, binds to the promoter regions of the target genes, and induces active transcription of inflammatory genes [10,11].

The precise molecular mechanisms responsible for the anti-inflammatory effects of GSPE remain unclear. Both IL-1β and TNF-α have been suggested to be important mediators involved in the initiation and perpetuation of colonic inflammation in IBD [1,12]. And NF-*κ*B activation is the most critical step for IL-1β and TNF-α gene transcription. Activation of NF-*κ*B may be a pivotal event in pro-inflammatory signal transduction [10,13]. Thus, we hypothesized that GSPE suppresses inflammatory responses that are possibly associated with the expression of NF-*κ*B. In order to elucidate the mechanisms, the expression of the NF-*κ*B and I*κ*B systems have been measured [1].

In this study, we also determined IL-1β and MPO. The results further document that GSPE significantly reduces: (1) the degree of colonic injury; (2) neutrophil infiltration and oxidative damage; (3) the release of proinflammatory cytokine IL-1β; (4) NF-*κ*B expression caused by TNBS in the colon. The different doses of GSPE showed significantly dose-dependent effects on I*κ*K levels in TNBS-induced rat colitis. However, GSPE did not show dose-dependent effects on MPO, IL-1β, PI*κ*Bα and NF-*κ*B levels. The cause may be that the low dose of GSPE or medium dose of GSPE shows maximum therapeutic effect, so the effect is not increase obviously along with the increase in other doses.

These results suggest that oral administration of GSPE effectively suppresses mucosal inflammation in the colon through the inhibition of NF-*κB* signal transduction pathways. Thus, it is possible to consider that GSPE suppresses I*κ*K activation, and the inactivated I*κ*K complex suppresses the phosphorylation-induced degradation of I*κ*Bα. Therefore the NF-*κ*B signal can be abrogated by newly-synthesized I*κ*Bα which enters the nucleus, removes NF-*κ*B dimers from DNA, and results in their exportin-mediated transport to the cytoplasm. Inhibition of NF-*κ*B signal transduction pathways may be one of the major mechanisms underlying the prevention of the development of TNBS-induced colitis. Thus, the inhibitory effect of GSPE on the development of TNBS-induced colitis was associated with the blockade of NF-*κ*B in the colon tissue.

Some studies show that IL-1β and hydroxyl radical can activate NF-*κ*B, and likewise, activated NF-*κ*B would be up-regulating proinflammatory target genes for cytokines production and oxidative damage [14]. It was reported that IL-1β and oxidative damage are significantly increase as well as the expression of NF-*κ*B in colonic tissue with colonitis. This could have attracted more neutrophils on the site of inflammation, further increasing the generation of hydroxyl radical and the damage to surrounding tissues [15,16,17]. It has been well demonstrated in our study that IL-1β elevation, oxidative damage and neutrophil infiltration are involved in the pathogenesis of colitis as they are present in the colon tissues and can be detected in the inflamed tissues. The decrease of the colonic inflammation, oxidative damage, IL-1β and NF-*κ*B demonstrated in current study expands possible mechanisms for the good effect of GSPE observed. The inhibition of the oxidative damage, IL-1β production and neutrophil infiltration by GSPE observed in current study is most likely attributed to the inhibitory effect on the activation of NF-*κ*B. In addition, the inactivated NF-*κ*B would be down-regulating IL-1β, neutrophil infiltration and oxidative damage.

## 3. Experimental

### 3.1. Animals

Male Wistar rats, 9–10 weeks of age and 180~200 g upon arrival, were obtained from the Shanghai SLAC Laboratory Animal Company. The rats were housed in plastic cages in a room kept at a constant temperature of 22 ± 2 °C, and they had free access to tap water and food throughout the study. Rats were feed with blocks of chow (14%–16% protein) supplied by the Laboratory Animal Center of Lanzhou University. The housing and handling of the experimental animals were in accordance with the guidelines of the Chinese Council for Animal Care.

### 3.2. Drug and Reagents

Proanthocyanidins from grape seeds (GSPE) was obtained from Tianjin Jianfeng Natural Products Company. Sulfasalazine (SASP) was obtained from Shanghai Sine Jiahua Pharmaceutical. TNBS (5%, *w/v*) was obtained from Sigma. ELISA kits of rat phospho-I kappaB-alpha (pI*κ*Bα), inhibitor kappa B kinase (I*κ*K), were all purchased from USCN life Science & Technology company (Wuhan, China). Trans-AM NF-*κ*B ELISA kits purchased from Active Motif (Carlsbad, CA, USA). Kit of IL-1β was purchased from Bender (Germany). Kit of Myeloperoxidase (MPO) was purchased from Nanjing Jiancheng Bioengineering Institute (Nanjing, China).

### 3.3. Drug and Agent Preparation

Low, medium, and high (10, 20, and 40 mg) doses of GSPE and 50 mg of SASP was each suspended in 1 mL of physiological saline; 25 mg of TNBS was dissolved in 1 mL of 50% ethanol (*v/v*).

### 3.4. Induction of Experimental UC and Treatment Protocols

Rats were randomly divided into seven groups: Normal control (n = 12), 50% ethanol control (n = 12), TNBS control (n = 14), SASP (n = 12) as a positive control, and the 3 GSPE treatment groups (each n = 14), which consisted of low dose (GSPE-L, 100 mg·kg^−1^), medium dose (GSPE-M, 200 mg·kg^−1^), and high dose (GSPE-H, 400 mg·kg^−1^).

### 3.5. Induction of UC

To induce colitis, rats were fasted for 48 h, and then anaesthetized with sodium pentobarbital (40 mg·kg^−1^ i.p.). An obtuse cannula was inserted into the anus of the rats and the tip was advanced approximately 8 cm. TNBS (100 mg·kg^−1^) was instilled into the colon through the cannula. After the instillation of the TNBS solution, the rats were kept in a head-down position for 30 min to prevent leakage of the intracolonic instillation. The rats from the normal control group and 50% ethanol control group were respectively instilled with physiologic saline or 50% ethanol at 4 mL·kg^−1^[18].

### 3.6. Treatment

Twenty-four hours after induction of UC, treatment began and was continued for seven days. Rats in the SASP and GSPE groups were given SASP or GSPE intragastrically, respectively; rats in the TNBS control group, the normal control group and 50% ethanol control group were administrated physiological saline intragastrically. The dosage of SASP or GSPE was 1 mL/100 g, once daily, for a total of seven days. These doses were chosen on the basis of a previous study, as they were the most active in preventing the TNBS-induced colitis in rats [7]. Then the rats were killed with an overdose of sodium pentobarbital.

### 3.7. Assessment of Colonic Damage and Acquisition of Specimens

The rats were anaesthetized with sodium pentobarbital (40 mg·kg^−1^ i.p.). The entire colon was excised and cleaned of adherent adipose tissue (removing the cecum and appendix), opened longitudinally, rinsed with cold physiological saline to remove fecal residue, then blotted, weighed. Representative colon specimens were taken from a region of the inflamed site adjacent to the macroscopic damage of each rat, and were fixed in 4% buffered formaldehyde. Equivalent colon segments were also obtained from the normal control group for microscopic evaluation. The formalin-fixed colon tissues were embedded in paraffin, and 5 μm sections were stained with hematoxylin and eosin (H&E). The microscopic damage was observed, such as ulceration and/or inflammation, as well as on the depth of the intestinal layer affected by the inflammatory process. Subsequently a region of the inflamed colon were divided longitudinally and accurately weight to determine biochemical markers. Subsequently, a region of the inflamed colon was divided longitudinally and accurately weighed to determine biochemical markers. One gram of colon tissues was homogenized in 9 mL of cold physiological saline. The homogenates were retained for measuring MPO activity. The remaining homogenates were centrifuged at 3,000 r/min and 4 °C for 10 min, the supernatants were transferred into several new tubes, frozen at −20 °C for measurements of IL-1β levels in tissues.

### 3.8. Preparation of Nuclear and Cytosolic Fractions

Colon tissues were homogenized in cold PBS, and then were centrifuged at 3,000 × g for 5 min. The resulting supernatants were discarded. The precipitates were washed two times with cold PBS and then resuspended in buffer A (10 mM HEPES buffer, pH 7.9, containing 10 mM KCl, 0.1 mM EDTA, 0.1 mM EGTA, 1mM dithiothreitol (DTT), 50 mM NaF, 30 mM β glycerophosphate, 1 mM Na_3_VO_4_ and 1 mM phenylmethylsulfonyl fluoride (PMSF)) and 10% NP-40, the homogenates were incubated for 15 min on ice and strong shocked for 45 s, then centrifuged at 15,000 × g for 15 min. The supernatants constituted cytosolic fractions, the supernatants were transferred into several new tubes, and frozen at −80 °C for measurements of pI*κ*Bα, I*κ*K. The pellets were washed three times with buffer A, then resuspended in buffer B (20 mM HEPES buffer, pH 7.9, 400 mM NaCl, 1 mM EDTA, 1 mM EGTA, 1 mM DTT and 1 mM PMSF) and shocked in 4 °C for 30 min, then centrifuged at 15,000 × g for 15 min. The supernatants were used as nuclear extracts, and they were frozen at −80 °C for measurements of NF-*κ*B.

### 3.9. Statistical Analysis

All results were expressed as mean ± SD. One-way ANOVA was used to test differences for single group analysis. Statistical significance was set at *P* < 0.05.

## 4. Conclusions

In conclusion, this study has demonstrated that the degree of colitis caused by TNBS was significantly attenuated by GSPE, which appears to stabilize I*κ*Bα against activation induced degradation and thereby reduce the amount of functionally active NF-*κ*B available in the nucleus. This effect of GSPE was associated with inhibition of NF-*κ*B signal transduction pathways and blockade of infiltration of inflammatory cells.
